# Design of a Phase I Drug Combination Study with Adaptive Allocation Based on Dose-Limiting Toxicity Attribution

**DOI:** 10.3390/cancers17061038

**Published:** 2025-03-20

**Authors:** Nolan A. Wages, Bethany J. Horton, Li Liu, Enrica Marchi, Gina R. Petroni

**Affiliations:** 1 Department of Biostatistics, Virginia Commonwealth University, Richmond, VA 23284, USA; 2 Massey Comprehensive Cancer Center, Richmond, VA 23298, USA; 3 Division of Translational Research & Applied Statistics, Department of Public Health Sciences, University of Virginia, Charlottesville, VA 22908, USA; 4 Division of Hematology/Oncology, Department of Medicine, University of Virginia NCI-Designated Cancer Center, Charlottesville, VA 23298, USA; em5yt@uvahealth.org

**Keywords:** dose finding, partial order continual reassessment method, drug combinations, toxicity attribution, early-phase, cancer

## Abstract

**Background:** This article describes the adaptation of a Phase I drug combination method to incorporate dose-limiting toxicity (DLT) attribution in dose assignments. The study is motivated by the Embolden trial (NCT03240211), a Phase Ib, multicenter trial at the UVA Comprehensive Cancer Center evaluating pembrolizumab with pralatrexate (Arm A), decitabine (Arm C), or both (Arm B) in relapsed/refractory peripheral and cutaneous T cell lymphomas. **Methods:** While Arms A and C used monotherapy dose escalation, Arm B required simultaneous escalation of both agents, integrating drug-specific DLT attribution to guide dosing. **Results:** We adapted the partial order continual reassessment method (POCRM) to incorporate this attribution, ensuring appropriate de-escalation of the offending agent. Given the trial’s complexity, software modifications were necessary to evaluate design performance through simulations. **Conclusions:** This work underscores the importance of novel dose-finding strategies in early-phase trials and aims to promote their broader adoption for improved trial efficiency and transparency.

## Simple Summary

Determining the right dose of drug combinations in early-phase cancer trials is challenging, especially when it is unclear which drug is causing side effects. This study introduces a novel dose-finding approach used in the EMBOLDEN trial, which tested the combination of pembrolizumab and pralatrexate in patients with relapsed or refractory T cell lymphomas. The method incorporates dose-limiting toxicity (DLT) attribution, allowing researchers to identify which drug is responsible for side effects and adjust dosing accordingly. By adapting the partial order continual reassessment method (POCRM), the design ensures safer and more accurate dose escalation. This innovative approach aims to improve the efficiency and transparency of dose-finding in early-phase clinical trials, ultimately enhancing patient safety and treatment effectiveness.

## 1. Introduction

This article describes the design of an early-phase drug combination study in which one of the agents of the combination is assumed to be associated with specific types of adverse events, whereas the other agent is less likely to cause severe toxicity. Herein, we describe the adaptation of a model-based Phase I drug combination method to incorporate the attribution of an observed dose-limiting toxicity (DLT) to one of the agents being studied to adaptively assign participants to candidate dose combinations. The motivating example for this study is the Embolden trial (NCT03240211), a Phase Ib, multicenter, prospective trial designed at the University of Virginia (UVA) Comprehensive Cancer Center. The trial evaluates the safety and tolerability of pembrolizumab combined with pralatrexate (Arm A), decitabine (Arm C), or both pralatrexate and decitabine (Arm B) in patients with relapsed/refractory (R/R) peripheral T cell lymphoma (PTCL) and cutaneous T cell lymphoma (CTCL). All arms administer a fixed dose of pembrolizumab alongside the other agents. Participants were enrolled into the study in the following order Arm A → Arm C → Arm B until the MTD for each arm is identified. In Arms A and C, dose escalation is limited to a single agent—pralatrexate in Arm A and decitabine in Arm C—using a classic dose escalation design suited for monotherapy dose escalation. Since data from these arms did not inform the development of Arm B, their details are omitted. This paper focuses on the design of Arm B, which involves the simultaneous escalation of both pralatrexate and decitabine, incorporating drug-specific adverse event attribution to guide dose assignment. From prior experience with the study drugs, DLTs are attributed to specific agents based on the type of observed toxicity. If an observed DLT is mucositis, thrombocytopenia, or a combination of mucositis with neutropenia and/or thrombocytopenia, it is assumed to be attributable to pralatrexate. Conversely, if an observed DLT is neutropenia alone or in combination with thrombocytopenia, it is assumed to be attributable to decitabine. For ethical reasons, if a participant has a DLT attributable to one of the study agents when treated at dose level d of that agent, then d should not be increased for that agent when the next participant cohort enters the trial.

Few methods have been developed to incorporate dose-limiting toxicity (DLT) attribution into dose assignment. Yin and Yuan [1] proposed a design using a copula model, specifically the Gumbel model [2], to estimate the joint probability of toxicity. This model allows investigators to compute DLT probabilities when toxicity is attributed to one drug, the other, or both. However, it assumes all toxicities are attributable, which is uncommon in practice. Wheeler et al. [3] introduced a semi-attributable toxicity design for trials with non-concurrent drug administration, where one drug is administered at the start of a treatment cycle and the second is introduced later only if no DLT occurs. If a DLT arises before the second drug is administered, it is attributed to the first drug. If it occurs afterward, attribution becomes uncertain. Iasonos and O’Quigley [4] proposed a monotherapy evaluation method that reduces bias from attribution errors by using personalized scores instead of binary DLT outcomes. Lee and Fan [5] addressed toxicity attribution in rule-based designs with non-overlapping toxicities. Despite these advancements, there remains a critical need for new methodologies that better address toxicity attribution, allowing for more flexible and accurate dose-finding in multi-agent trials.

This paper describes a design that adapts the partial order continual reassessment method (POCRM) [6] to incorporate DLT attribution into dose assignment. While the POCRM is used throughout the study to estimate DLT probabilities, modifications were necessary to accommodate the investigators’ requirement to de-escalate the drug attributed to a DLT. Adjustments to software were made to evaluate specific trial aspects, such as simulating different DLT probability scenarios based on the type of DLT observed. As a result, direct application of the POCRM was not feasible. This trial contributes to a growing number of studies that have modified the POCRM to address unique challenges in modern early-phase trials [7,8,9,10,11].

The key advantage of this model-based approach over traditional rule-based methods is its ability to adapt to accumulating toxicity data. After each participant’s enrollment, the model updates DLT probability estimates and refines dose recommendations based on toxicity attribution. This allows for more efficient dose escalation and de-escalation decisions, potentially reducing the number of participants treated at sub-therapeutic dose combinations. Unlike treatment details, study designs are typically not available on public registries like ClinicalTrials.gov, leading to a lack of transparency that hinders the implementation of novel designs. This manuscript aims to highlight published examples of innovative trial designs, demonstrating the flexibility of adaptive methods in meeting evolving trial needs. Increasing the visibility of such designs can help to overcome the barriers to their adoption in early-phase trials.

Additionally, given the lengthy timeline from study conception to protocol completion, it is important to present design considerations with broad applicability. Even after study completion, journals rarely require full protocols for dose-finding trials, and final publications often lack space to describe novel designs in detail. Thus, it is important to communicate that innovative methods are actively being used in clinical practice. The next section outlines the statistical considerations for designing Arm B of the Embolden trial, followed by simulation studies evaluating the design’s operating characteristics. The manuscript concludes with a discussion of the implementation of the proposed design and potential directions for future research.

## 2. Methods

### 2.1. Design Considerations

**Figure 1:**
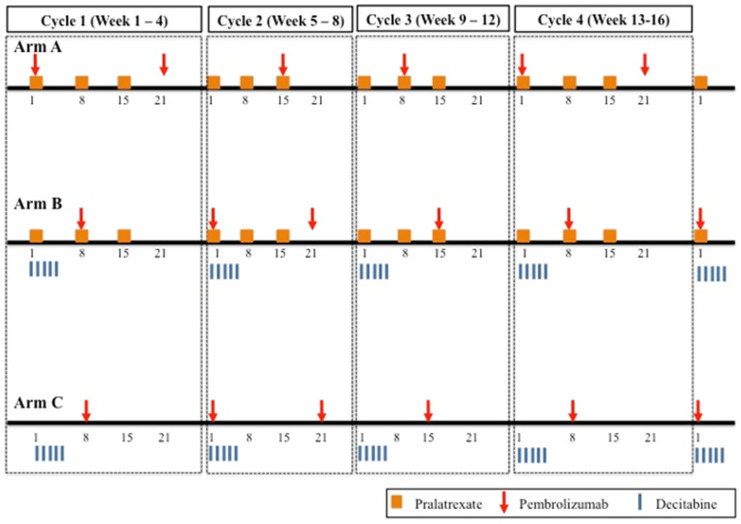
Embolden (NCT03240211) trial schema.

Arm B of the Embolden (NCT03240211) trial is an early-phase evaluation of the safety and tolerability of the combination of five dosing schedules (15 mg days 1 and 15; 20 mg days 1 and 15; 20 mg days 1, 8, and 15; 30 mg days 1 and 15; 30 mg days 1, 8, and 15) of pralatrexate, with three dosing schedules of decitabine (10 mg days 1–3; 10 mg days 1–5; 20 mg days 1–3) (Table 1 and Figure 1). The trial was initially opened at Columbia University using a rule-based approach for Arm B that would explore only a subset of the fifteen total combinations. Arm B accrued four total participants before the principal investigators relocated to UVA. Discussions between statisticians and clinical investigators at UVA lead to a consensus that all combinations were worth exploring and that a model-based design strategy was the most efficient use of trial resources. Fixing the escalation path to prespecified combinations restricts the number of dose combinations that can be explored, potentially overlooking promising options outside the predefined path. This trial aims to identify the maximum tolerated dose combination (MTDC), defined as the combination with a DLT rate closest to the target of 25%. The study is currently open to accrual, and the first participant was enrolled at UVA under the new Arm B design in May 2024. To monitor safety, adverse events are assessed, and acute toxicities are graded using the National Cancer Institute (NCI) Common Terminology Criteria for Adverse Events (CTCAE) Version 5. As data accumulate, each participant is classified as experiencing a DLT (yes/no) based on protocol-defined criteria observed during the first treatment cycle. Based on the accumulated data in Arm B from 1 observed DLT in 4 previous participants accrued to Combination 8 (i.e., Pralatrexate 20 mg [days 1, 8, and 15] and Decitabine 10 mg [days 1–5]) at Columbia University, the 1st eligible participant was entered onto Combination 8 (Table 1).

### 2.2. Estimation

Safety assessments assume that increasing the dose of one agent while holding the other fixed leads to a higher probability of DLT. In other words, DLT probabilities increase across rows and up columns in the drug combination grid (Table 1). However, the relative toxicities of combinations along the same diagonal in Table 1 are uncertain—for example, Combination 2 may have a higher or lower true DLT probability than Combination 3. To address this uncertainty, we specify multiple one-parameter regression models representing different possible orderings of DLT probabilities (Table 2). Model selection techniques are then used to identify the model that best fits the data [6]. A common approach in the continual reassessment method (CRM [12]) is to raise a set of prespecified constants—known as the ‘skeleton’ (Table 3)—to a power exp (a), where a is a parameter to be estimated by the data [13]. Skeleton values for each model were generated using the Lee and Cheung algorithm [14] to ensure robust operating characteristics across a range of scenarios. Within each ordering, the CRM estimates DLT probabilities using the accumulated data. For each possible ordering, m=1,⋯, 6 in Table 2, DLT probabilities are modeled using a one-parameter power model: $R\left(d_{i}\right)=P\;r\left(\mathrm{DLT}\;\mathrm{at}\;\mathrm{combination}\;i\;\right)\approx p_{mi}^{{\exp\;(\theta }_{m})},$ where $p_{mi}$ represents the skeleton values for ordering m from Table 3. Each ordering is assigned equal prior weight $\tau \left(m\right)=1/m$. After each participant is accrued, the parameter $\theta_{m}$ is estimated for each ordering via maximum likelihood estimation using the likelihood function

$$L_{m}\left(\theta_{m}\right)=\prod_{i=1}^{15}{\left(p_{mi}^{{\exp\;(\theta }_{m})}\right)}^{y_{i}}{\left(1-p_{mi}^{{\exp\;(\theta }_{m})}\right)}^{{n_{i}-y}_{i}},$$

where $y_{i}\;$= the number of DLTs, and $n_{i}\;$= the number of treated participants in combination i. The ordering h with the highest likelihood is selected and, within this ordering, DLT probability estimates are updated. If multiple orderings have the same likelihood, one is chosen randomly. After each cohort is accrued, the DLT probability estimates $\tilde{R}(d_{i})$ are updated accordingly:

$$\tilde{R}\left(d_{i}\right)=p_{hi}^{\exp\left({\tilde{\theta }}_{h}\right)};\;{\tilde{\theta }}_{h}=\mathrm{arg max}\;L_{h}\left(\theta_{h}\right)$$

The DLT probability estimates are used to sequentially allocate participants according to the following algorithm.

### 2.3. Allocation

1. If the most recent participant accrued to the study does not experience a DLT, then the recommended combination for the next participant will be the combination indicated by the model to have an estimated DLT rate closest to 25%. Escalation is restricted to adjacent dose combinations that differ from the current combination by one dose level of one drug.
2. If the most recent participant accrued to the study does experience a DLT, then attribution by study agent occurs such that
  - If the DLT is mucositis or thrombocytopenia or mucositis plus neutropenia and/or thrombocytopenia, then the recommended combination for the next participant will be restricted to either the current combination administered to the most recent participant or to de-escalation by one dose level of pralatrexate, based on which combination has an estimated DLT rate closest to 25%. We designate the occurrence of this event as a “Type 1” DLT.
  - If the DLT is neutropenia or neutropenia and thrombocytopenia, then the recommended combination for the next participant will be restricted to either the current combination administered to the most recent participant or to de-escalation by one dose level of decitabine, based on which combination has an estimated DLT rate closest to 25%. We designate the occurrence of this event as a “Type 2” DLT.
  - If the DLT is neither a Type 1 nor Type 2 event, we designate it as a “Type 3” DLT event. If Type 1 and Type 2 events occur simultaneously, then the attribution cannot be ascertained and the event is considered to be a Type 3 event.

The conduct of Arm B will sequentially repeat the estimation procedure after the enrollment and follow-up of each participant (i.e., cohort size 1). The allocation procedure will be repeated until sufficient information about the MTDC has been obtained according to the stopping rules described below.

### 2.4. Stopping the Trial

Accrual to Arm B will be paused for review by the study investigators and the Data Safety and Monitoring Committee. They will assess whether accrual should be modified or permanently closed based on the following criteria:

1. Accrual to Arm B will be stopped for safety if the observed DLT rate at the lowest combination (Combination 1) is ≥ the number of DLTs out of the number of participants treated at the lowest combination as displayed in Table 4. The stopping guidelines in Table 4 are based on whether the lower limit of an Agresti–Coull [15] binomial confidence interval (with 80% confidence) for the lowest combination exceeds the target DLT rate. The bounds were generated using the web application at http://uvatrapps.shinyapps.io/pocrm/, accessed on 20 October 2020.

2. If the recommendation is to assign the next participant to a combination that already has 10 participants (including the four Columbia participants) treated on the combination, accrual to Arm B will be stopped and the recommended combination is declared the MTDC.
3. Otherwise, the MTDC is defined as the combination that is recommended after the maximum sample size of 30 participants are accrued to the study.

A maximum target accrual of 26 additional eligible participants is planned in Arm B (total accrual n = 30), but the simulation results below indicate that the estimated sample size needed to complete accrual is approximately 18 to 23 participants. Additional participants may be enrolled to replace any participants who are enrolled but do not receive treatment. Adjusting for an approximate 5% drop-out/ineligibility rate, maximum accrual should not exceed 30 participants.

## 3. Results

### 3.1. Simulation Studies to Evaluate the Design

To assess the performance and robustness of the proposed design, we conducted comprehensive simulation studies. These simulations aimed to evaluate the design’s ability to identify the MTDC and explore its operating characteristics under various scenarios. The studies included two components: (1) a single simulated trial and (2) operating characteristics derived from extensive simulations across multiple scenarios.

#### 3.1.1. Single Simulated Trial Illustration

A single simulated trial was conducted to illustrate the trial’s progression and the adaptive decision-making process, using the true dose-limiting toxicity (DLT) probabilities specified in Scenario 4 of our simulation studies (Supplemental Table S4). In this scenario, Combinations 4 and 5 are identified as the true MTDCs, as both have true DLT probabilities of 0.25, matching the target DLT rate that defines the MTDC. The trial planned to enroll a maximum of 30 participants sequentially in cohorts of size 1, with dose escalation and de-escalation decisions guided by the model’s recommendations and trial termination guided by the stopping rules described above. The simulation used the model defined by the skeleton values in Table 3 and targeted a DLT rate of 0.25 to define the MTDC. The initial dose combination selected was Combination 8, with the trial stopping if at least 10 participants were treated at one combination or if the confidence level for the stopping rule exceeded 0.8. As participants were treated, the design adapted the dose combination assignment based on the observed outcomes. Table 5 illustrates the simulated participant-specific outcomes and dose combination assignments after each participant was enrolled. The DLT outcomes for each participant (“1” for experiencing a DLT and “0” for no DLT) and the type of DLT observed (“Type 1”, “Type 2”, or “Type 3” as defined above) are recorded.

The simulated DLT outcomes and adaptive dose assignments are detailed in Table 5, demonstrating the design’s dynamic learning capability. For example, after the 2nd participant, a DLT of Type 1 is observed, indicating that the dose of pralatrexate should be de-escalated if the assignment algorithm recommends de-escalation. The trial de-escalates from Combination 11 to Combination 8, corresponding to a reduction in the dose of pralatrexate. After the 24th participant, the model correctly recommended Combination 4 as the MTDC, as it had already been administered to n = 10 participants throughout the study and was recommended for the 25th participant. The trial’s trajectory showcases the design’s ability to adaptively refine its dose-selection process while maintaining participant safety.

#### 3.1.2. Operating Characteristics

Operating characteristics were evaluated through the simulation of 5000 trials under various scenarios, each defined by a different set of true DLT probabilities. These scenarios were chosen to represent plausible clinical settings, including varying degrees of dose–toxicity relationships and distributions of DLT probabilities across combinations. Each scenario targeted a DLT rate of 0.25 to define the MTDC. Scenario-specific true DLT probabilities are detailed in Supplemental Tables S1–S5.

Key metrics evaluated included the probability of correct selection (PCS) of the MTDC, which represents the percentage of trials correctly identifying the true MTDC at the study’s conclusion, the average number of observed DLTs per trial, the average sample size, the number of participants treated at the MTDC, and the percentage of trials terminated early due to safety concerns. Scenario 1 assumed a relatively uniform increase in DLT probabilities across combinations. The design achieved a PCS of 38.9%, with an average of 5.0 DLTs observed per trial. The average sample size was 22.8, and 6.8 participants, on average, were treated at MTDCs. Early stopping for safety were negligible in number (0%).

Scenario 2 featured a steeper dose–toxicity relationship. The PCS improved to 67.9%, reflecting the design’s ability to differentiate between combinations with distinct safety profiles. An average of 6.1 DLTs were observed per trial. The average sample size remained consistent (21.6), with 11.3 participants, on average, treated at true MTDCs. Early stopping occurred in 0% of trials. In Scenario 3, true DLT probabilities were lower overall. The PCS was 38.9%, with an average 4.0 observed DLTs per trial. The average sample size was 22.91, and 4.8 participants, on average, were treated at the MTDC. No early terminations occurred. Scenario 4 involved higher overall DLT probabilities, posing a greater challenge for safety. The PCS was 42.7%, with an average of 8.0 observed DLTs per trial. Early stopping increased to 0.1%, highlighting the design’s protective measures in high-risk settings. The average sample size was 23.9 participants, with an average of 6.9 participants treated at MTDCs. Scenario 5 represented extreme toxicity probabilities. The PCS was 48.7%, the highest among all scenarios. However, the design showed an average of 9.3 DLTs per trial, reflecting the inherent risk in this setting. The average sample size was 24.8 participants, with an average of 7.0 participants treated at MTDCs. Early stopping reached 0.6%, underscoring the importance of safety monitoring in such scenarios.

Table 6 summarizes the operating characteristics for each scenario, while more detailed simulation results are provided in the supplemental material. These results demonstrate the design’s robustness and adaptability across diverse clinical contexts. The PCS and average sample size metrics confirm the design’s efficiency in identifying the MTDC while minimizing participant exposure to excessively toxic combinations. The simulation studies validate the proposed early-phase trial design’s ability to dynamically adapt to accumulating data. The design achieves a high probability of selecting the correct MTDC with manageable sample sizes while maintaining participant safety through sensible dose escalation decisions and early stopping rules. These findings support the design’s applicability to a broad range of clinical trial settings.

## 4. Conclusions

The proposed design represents an advancement in dose-finding strategies for early-phase combination trials by incorporating DLT attribution into the POCRM modeling framework. By leveraging a flexible model-based framework, this design addresses challenges posed by the complex nature of toxicity attribution in multi-agent settings, offering a practical solution for ethical and efficient dose allocation. Simulation results demonstrate its robustness in identifying the MTDC while minimizing participant exposure to unsafe dose levels. The adaptive nature of the design ensures that trial recommendations are continually refined based on accumulating data, thereby improving the reliability and safety of dose recommendations.

Despite the rapid development of novel early-phase dose-finding methods over the past decade, the adoption of innovative designs in practice remains limited [16,17,18,19]. Regulatory bodies such as the FDA have increasingly encouraged the use of novel approaches [20,21,22,23,24,25,26]. This work underscores the value of sharing detailed methodologies and practical adaptations of novel designs to promote transparency and innovation in early-phase clinical trials. As the complexity of therapeutic combinations increases, methods like the one described here will be critical for optimizing dose selection and accelerating the development of safe and effective treatments. The numerical results from the simulation studies exemplify the type of evidence that enhances understanding, acceptance, and regulatory approval of novel designs [27,28,29,30,31]. Such support for adaptive methods will contribute to more efficient trial designs in contemporary dose-finding studies, with the potential to significantly impact the future of oncology care [32,33,34]. Future research could explore extending this framework to more complex modeling frameworks that integrate additional endpoints, such as efficacy or patient-reported outcomes, further enhancing its applicability to modern oncology trials.

## Figures and Tables

**Table 1:** Combination designation for Arm B of the Embolden trial (NCT03240211).

Pralatrexate	30 mg days 1, 8, and 15	Combination 12	Combination 14	Combination 15
	30 mg days 1 and 15	Combination 9	Combination 11	Combination 13
	20 mg days 1, 8, and 15	Combination 6	Combination 8	Combination 10
	20 mg days 1 and 15	Combination 3	Combination 5	Combination 7
	15 mg days 1 and 15	Combination 1	Combination 2	Combination 4
		10 mg days 1–3	10 mg days 1–5	20 mg days 1–3
		Decitabine		

**Table 2:** Possible orderings of DLT probabilities. The values in this table represent the number of the drug combination from Table 1 (i.e., Combination 1 is represented by “1”, Combination 2 is represented by “2”, etc.

(1) 1-2-3-4-5-6-7-8-9-10-11-12-13-14-15
(2) 1-2-3-6-5-4-7-8-9-12-11-10-13-14-15
(3) 1-3-6-9-12-2-5-8-11-14-4-7-10-13-15
(4) 1-2-4-3-5-7-6-8-10-9-11-13-12-14-15
(5) 1-3-2-6-5-4-9-8-7-12-11-10-14-13-15
(6) 1-3-2-4-5-6-9-8-7-10-11-12-14-13-15

**Table 3:** Skeleton of DLT probabilities under each ordering. Skeleton values were chosen according to the algorithm of Lee and Cheung [14].

	Combination Labels														
Order	1	2	3	4	5	6	7	8	9	10	11	12	13	14	15
1	0.001	0.004	0.010	0.03	0.06	0.11	0.17	0.25	0.33	0.42	0.50	0.58	0.65	0.71	0.76
2	0.001	0.004	0.010	0.11	0.06	0.03	0.17	0.25	0.33	0.58	0.50	0.42	0.65	0.71	0.76
3	0.001	0.110	0.004	0.50	0.17	0.01	0.58	0.25	0.03	0.65	0.33	0.06	0.71	0.42	0.76
4	0.001	0.004	0.030	0.01	0.06	0.17	0.11	0.25	0.42	0.33	0.50	0.65	0.58	0.71	0.76
5	0.001	0.010	0.004	0.11	0.06	0.03	0.33	0.25	0.17	0.58	0.50	0.42	0.71	0.65	0.76
6	0.001	0.010	0.004	0.03	0.06	0.11	0.33	0.25	0.17	0.42	0.50	0.58	0.71	0.65	0.76

**Table 4:** Stopping guidelines for DLTs at the lowest combination.

Number of Participants	Boundary
2–3	≥2
4–6	≥3
7–9	≥4
10	≥5

**Table 5:** A simulated sequential trial illustrating the described design. The maximum tolerated dose combination (MTDC) recommendation is Combination 10 after 19 participants. DLT Outcome: 1 = the participant experienced a DLT; 0 = no DLT was observed. DLT Type: Type 1 = deescalate pralatrexate or stay at the current dose pair; Type 2 = deescalate decitabine or stay at the current dose pair; Type 3 = the DLT is neither a Type 1 nor Type 2 event. If Type 1 and Type 2 events occur simultaneously, then the attribution cannot be ascertained and the event is considered to be a Type 3 event.

Participant ID	Dose Pair Assigned	True DLT Rate of Assigned Dose Pair	DLT Outcome	DLT Type
1	8	0.47	0	-
2	11	0.56	1	1
3	8	0.47	1	2
4	8	0.47	1	3
5	5	0.25	0	-
6	4	0.25	0	-
7	7	0.47	1	-
8	4	0.25	1	-
9	4	0.25	0	-
10	5	0.25	1	2
11	5	0.25	1	1
12	2	0.18	0	-
13	2	0.18	0	-
14	4	0.25	0	-
15	4	0.25	1	1
16	4	0.25	0	-
17	4	0.25	1	1
18	4	0.25	0	-
19	5	0.25	1	2
20	3	0.18	0	-
21	2	0.18	0	-
22	2	0.18	0	-
23	4	0.25	0	-
24	4	0.25	0	-

**Table 6:** Summary of the simulation studies evaluating the design operating characteristics. PCS = percentage of correct selection of the MTDC.

	Scenario 1	Scenario 2	Scenario 3	Scenario 4	Scenario 5
PCS	38.9	67.9	38.9	42.7	48.7
# of observed DLTs	5.0	6.1	4.0	8.0	9.3
# of average sample size	22.8	21.6	22.9	23.9	24.8
# of participants treated at MTDC	6.8	11.3	4.8	6.9	7.0
% of early stopping for safety	0.0	0.0	0.0	0.1	0.6

## Data Availability

This article presents the proposal and evaluation of new clinical trial designs using computer-generated (simulated) clinical trial outcome data. No real patient data were analyzed in this study.

## Supplementary Materials

The following supporting information can be downloaded at https://www.mdpi.com/article/10.3390/cancers17061038/s1, Table S1. Operating Characteristics for Scenario 1; Table S2. Operating Characteristics for Scenario 2; Table S3. Operating Characteristics for Scenario 3; Table S4. Operating Characteristics for Scenario 4; Table S5: Operating Characteristics for Scenario 5.
