# Supplementary material for: Design of a Phase I Drug Combination Study with Adaptive Allocation Based on Dose-Limiting Toxicity Attribution
Source: Cancers (Basel). 2025 Mar 20;17(6):1038. doi: 10.3390/cancers17061038 (PMC11940839; doi:10.3390/cancers17061038)
Supplement: Supplementary file 1 [file cancers-17-01038-s001.zip › cancers-3481299-supplementary.pdf]

Supplemental Material for "Design of a phase I drug combination study with adaptive allocation based on dose-limiting toxicity attribution"

Simulation settings:
target toxicity rate = 0.25
cohort size = 1
number of cohorts in a single trial = 30
number of simulated trials = 5000
number of patients needed on one combination to stop the trial = 10
starting combination = 8
confidence level used in stopping rule = 0.8
probability of adverse event A conditional on having a DLT (de-escalate Pralatrexate) = 0.2
probability of adverse event B conditional on having a DLT (de-escalate Decitabine) = 0.3
Skeleton values: 0.001, 0.004, 0.01, 0.03, 0.06, 0.11, 0.17, 0.25, 0.33, 0.42, 0.50, 0.58, 0.65, 0.71, 0.76
(skeleton values generated via R function getprior(0.04, 0.25, 8, 15))

Table S1: Operating Characteristics for Scenario 1

**True DLT probabilities**				
**Pralatrexate**	30mg 1,8,15	0.16	0.37	0.48
	30mg 1,15	0.15	**0.25**	0.36
	20mg 1,8,15	0.15	0.15	**0.25**
	20mg 1,15	0.09	0.14	0.15
	15mg 1,15	0.07	0.07	0.12
**MTD selection percentage**				
**Pralatrexate**	30mg 1,8,15	7.4	11.4	0.0
	30mg 1,15	5.0	**26.5**	1.8
	20mg 1,8,15	0.8	25.9	**12.4**
	20mg 1,15	0.1	1.5	6.0
	15mg 1,15	0.0	0.3	1.1
**Average number of patients treated**				
**Pralatrexate**	30mg 1,8,15	1.2	2.0	0.0
	30mg 1,15	1.8	**4.1**	0.3
	20mg 1,8,15	0.4	7.4	**2.7**
	20mg 1,15	0.0	0.6	1.9
	15mg 1,15	0.0	0.1	0.4
		10mg 1-3	10mg 1-5	20mg 1-3
		**Decitabine**		

Table S2: Operating Characteristics for Scenario 2

**True DLT probabilities**				
**Pralatrexate**	30mg 1,8,15	0.46	0.55	0.60
	30mg 1,15	0.38	0.45	0.50
	20mg 1,8,15	0.14	**0.25**	0.45
	20mg 1,15	0.1	0.14	**0.25**
	15mg 1,15	0.09	0.1	0.13
**MTD selection percentage**				
**Pralatrexate**	30mg 1,8,15	1.9	0.6	0.0
	30mg 1,15	9.2	3.4	0.0
	20mg 1,8,15	3.8	**57.3**	4.0
	20mg 1,15	0.1	4.5	**10.6**
	15mg 1,15	0.0	0.8	3.8
**Average number of patients treated**				
**Pralatrexate**	30mg 1,8,15	0.5	0.4	0.0
	30mg 1,15	2.5	1.4	0.0
	20mg 1,8,15	1.2	**8.7**	1.6
	20mg 1,15	0.1	1.5	**2.6**
	15mg 1,15	0.0	0.3	1.0
		10mg 1-3	10mg 1-5	20mg 1-3
		**Decitabine**		

Table S3: Operating Characteristics for Scenario 3

**True DLT probabilities**				
**Pralatrexate**	30mg 1,8,15	0.15	**0.25**	0.40
	30mg 1,15	0.11	0.14	**0.25**
	20mg 1,8,15	0.08	0.10	0.11
	20mg 1,15	0.06	0.07	0.08
	15mg 1,15	0.05	0.05	0.07
**MTD selection percentage**				
**Pralatrexate**	30mg 1,8,15	5.9	**27.7**	0.0
	30mg 1,15	2.1	28.4	**11.2**
	20mg 1,8,15	0.1	11.2	9.7
	20mg 1,15	0.0	0.1	2.8
	15mg 1,15	0.0	0.0	0.8
**Average number of patients treated**				
**Pralatrexate**	30mg 1,8,15	0.9	**3.4**	0.0
	30mg 1,15	1.1	5.3	**1.5**
	20mg 1,8,15	0.2	6.4	2.8
	20mg 1,15	0.0	0.2	1.1
	15mg 1,15	0.0	0.0	0.1
		10mg 1-3	10mg 1-5	20mg 1-3
		**Decitabine**		

Table S4: Operating Characteristics for Scenario 4

**True DLT probabilities**				
**Pralatrexate**	30mg 1,8,15	0.58	0.63	0.70
	30mg 1,15	0.49	0.56	0.62
	20mg 1,8,15	0.40	0.47	0.55
	20mg 1,15	0.18	**0.25**	0.47
	15mg 1,15	0.15	0.18	**0.25**
**MTD selection percentage**				
**Pralatrexate**	30mg 1,8,15	0.2	0.0	0.0
	30mg 1,15	0.4	0.2	0.0
	20mg 1,8,15	5.4	2.8	0.4
	20mg 1,15	4.1	**31.7**	23.6
	15mg 1,15	3.1	9.0	**11.0**
**Average number of patients treated**				
**Pralatrexate**	30mg 1,8,15	0.1	0.1	0.0
	30mg 1,15	1.2	0.5	0.0
	20mg 1,8,15	2.7	7.3	0.5
	20mg 1,15	0.9	**4.9**	2.0
	15mg 1,15	0.0	1.6	**2.0**
		10mg 1-3	10mg 1-5	20mg 1-3
		**Decitabine**		

Table S5: Operating Characteristics for Scenario 5

**True DLT probabilities**				
**Pralatrexate**	30mg 1,8,15	0.63	0.75	0.80
	30mg 1,15	0.56	0.62	0.70
	20mg 1,8,15	0.47	0.55	0.58
	20mg 1,15	**0.25**	0.47	0.49
	15mg 1,15	0.15	**0.25**	0.4
**MTD selection percentage**				
**Pralatrexate**	30mg 1,8,15	0.8	0.0	0.0
	30mg 1,15	1.2	0.0	0.0
	20mg 1,8,15	8.4	10.0	0.1
	20mg 1,15	**16.7**	16.3	1.7
	15mg 1,15	2.1	**32.1**	9.9
**Average number of patients treated**				
**Pralatrexate**	30mg 1,8,15	0.1	0.0	0.0
	30mg 1,15	0.8	0.3	0.0
	20mg 1,8,15	2.2	6.4	0.4
	20mg 1,15	**2.6**	4.4	1.1
	15mg 1,15	0.4	**4.4**	1.8
		10mg 1-3	10mg 1-5	20mg 1-3
		**Decitabine**		
